# Supplementary material for: Chromosome-length genome assembly of Uta stansburiana and gene expression data reveal fast pace-of-life comes with environmental stability
Source: bioRxiv. 2025 Jun 9:2025.05.28.656178. Preprint. [Version 3] doi: 10.1101/2025.05.28.656178 (PMC12154890; doi:10.1101/2025.05.28.656178)
Supplement: Supplement 1 [file media-1.zip › SuppFigs.docx]

**Supp. Mat. Fig 1. Sampling location with annual precipitation (mm)**

**Supp. Mat. Fig 2. Sampling location with average annual irradiation rate**

**Supp. Mat. Fig 3. Sampling locations with average annual UVB irradiation**

**Supp. Mat. Fig 4.** Heatmap of log2-transformed count data for genes with differential expression by region, controlling for season and sex. Samples are labeled by region, season, and sex. **B)** Principal components analysis (PC1, PC2) of regular log-transformed count data.

**
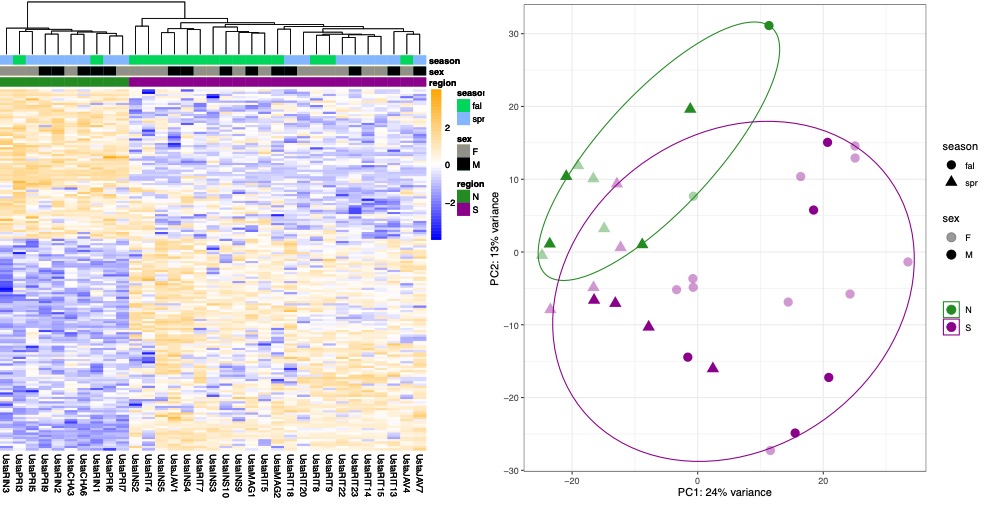
**

**Supp. Mat. Fig. 5. Volcano plot of genes differentially expressed between northern and southern groups, controlling for sex and seasonal differences. Positive log2 fold-change values correspond to genes differentially over-expressed in the southern group, while negative log2 fold-change values correspond to genes differentially under-expressed in the southern group.**

**Supp. Mat. Fig 6. Revigo treemap of enriched biological processes for the region controlling for season and sex contrast**

**Supp. Mat. 7. Revigo treemap of enriched biological processes for contrast by sex**

**Supp. Mat. 8. Distribution of scaffold size. Scaffold 1-17 correspond to HiC_scaffolds_1-17. HiC_scaffold_18 is likely the Y chromosome.**

**
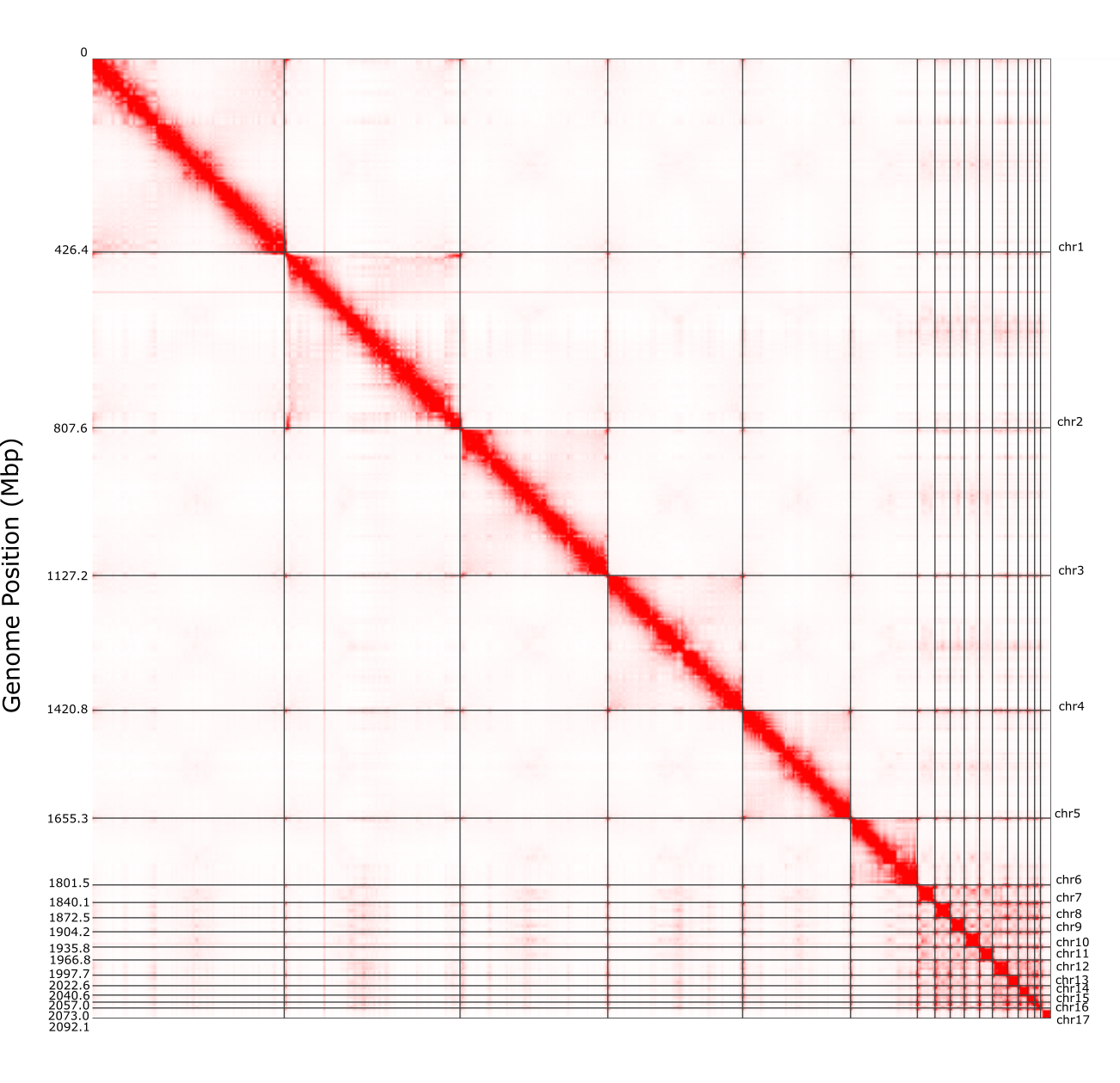
**

**Supp. Mat. 9. Interaction map of Hi-C chromosome-level data.**

**Supp. Mat. 10. Revigo treemap of enriched biological processes for yellow vs. orange- and blue-throated lizard contrast**

**Supp. Mat. 11. Volcano plot of genes differentially expressed between male and female lizards, controlling for regional and seasonal differences. Positive log2 fold-change values correspond to genes differentially over-expressed in males, while negative log2 fold-change values correspond to genes differentially under-expressed in the southern group (over-expressed in females).**

**Supp. Mat. 12. Volcano plot of genes differentially expressed between orange-throated and blue- and yellow-throated lizards. Positive log2 fold-change values correspond to genes differentially over-expressed in orange-throated lizards, while negative log2 fold-change values correspond to genes differentially under-expressed in the orange-throated lizards (over-expressed in blue- and yellow-throated lizards).**

**Supp. Mat. 12. Volcano plot of genes differentially expressed between blue-throated and orange- and yellow-throated lizards. Positive log2 fold-change values correspond to genes differentially over-expressed in blue-throated lizards, while negative log2 fold-change values correspond to genes differentially under-expressed in the blue-throated lizards (over-expressed in orange- and yellow-throated lizards).**

**Supp. Mat. 12. Volcano plot of genes differentially expressed between yellow-throated and blue- and orange-throated lizards. Positive log2 fold-change values correspond to genes differentially over-expressed in yellow-throated lizards, while negative log2 fold-change values correspond to genes differentially under-expressed in the yellow-throated lizards (over-expressed in blue- and orange-throated lizards).**
